# The Rome Criteria Divides, Distorts and Dilutes the Prevalence of Irritable Bowel Syndrome

**DOI:** 10.4103/1319-3767.65178

**Published:** 2010-07

**Authors:** Kok-Ann Gwee, Uday C. Ghoshal

**Affiliations:** Stomach Liver and Bowel Clinic, Gleneagles Hospital, Singapore; 1 Department of Gastroenterology, Sanjay Gandhi Postgraduate Institute of Medical Sciences, Lucknow, India

Functional bowel disorders (FBD) comprise a group of conditions that continue to challenge and defy clear definition and understanding. Patients experience a variable combination of gastrointestinal symptoms for which there is no organic basis that can be identified by routine medical tests. Functional bowel disorders are not life threatening, but its treatment is time consuming. As discussed in the paper from Iran by Sorouri *et al*, the reported prevalence worldwide is between 12 and 42%, and in Iran, FBDs make up 40% of gastroenterology outpatients.[1]

Therefore, it is surprising that in this well conducted study involving 18180 participants from Tehran province, Iran, the prevalence of the irritable bowel syndrome (IBS) (the most well studied of the FBD) was only 1.1%.[1] On the other hand, the prevalence of all FBDs, while modest, was still substantial at 10.9%. These numbers recall the low numbers reported in the study from Israel where the prevalence of IBS was only 2.9% when this was based on the Rome II criteria.[2] Another notable aspect of this new Iranian study was the high degree of overlap between FBD and functional dyspepsia; 83.8% of dyspepsia had FBD, and 68.5% of FBD had dyspepsia.[1] This situation is very similar to what we have observed in the rest of Asia.[3] We have observed that Asian patients may present with upper abdominal symptoms more often than Western population.[3–5] We have identified a number of studies from Asia, where it appeared that subjects with IBS may have been misclassified as suffering from dyspepsia.[3,6,7] In a study from Taiwan, more than half of patients initially classified as suffering from dyspepsia were reclassified as IBS when it was clarified that their upper abdominal pain was exclusively relieved with defecation.[6]

How do we make sense of all these numbers? In the study by Sperber *et al*, it was observed that a simple change in the definition allowing for milder symptoms to qualify for IBS, increased its prevalence almost four times in the same cohort, but the prevalence of FBD remained the same.[8] This suggests that the division of FBD into the various subsets is arbitrary, and in the process, produces distortion in the recognition of IBS. In the current Iranian study, there appears to be a similar distortion by the vagaries of the Rome criteria. Unspecified FBD (U-FBD) comprised the largest group of FBD, suggesting that there is a substantial group of subjects with FBD who just failed to meet the criteria for IBS. In U-FBD, bloating (64.4%) was more common than abdominal pain (46.7%).[1] U-FBD subjects also had the highest overlap with functional dyspepsia. We have argued that bloating should be recognized as one of the major symptoms of IBS, and it should also be recognized that IBS symptoms frequently occur after meals.[3] In a recent major Indian survey, the symptoms of IBS patients were studied unencumbered by the Rome criteria, and it was observed that abdominal fullness (bloating) was as common as abdominal pain (70% of 2785 patients).[4]

In their conclusions, the authors of this paper have appropriately highlighted issues with the Rome criteria itself.[1] It is timely that the Rome Foundation takes a more global perspective. To do so, more attention should be paid to the influence of culture on symptom reporting and therein, interpretation of gastrointestinal symptoms, and how this could affect the workup and diagnosis of functional GI diagnosis of FBDs worldwide. While the Rome criteria provide a useful yardstick for pharmaceutical industry and regulatory authorities, in our scientific exploration we should not homogenize for the sake of homogenizing. The underlying risk associated with this is losing potentially valuable clues that can help us to understand FBDs better. However, ultimately, it is still up to researchers in various parts of the world to stand up and be counted, as the authors of this Iranian study and others have recently done.[4]
